# Recent advances in understanding noroviruses

**DOI:** 10.12688/f1000research.10081.1

**Published:** 2017-01-26

**Authors:** Eric Bartnicki, Juliana Bragazzi Cunha, Abimbola O. Kolawole, Christiane E. Wobus

**Affiliations:** 1 Department of Microbiology and Immunology, University of Michigan Medical School, Ann Arbor, MI, USA

**Keywords:** RNA virus, Calicivirus, Norovirus, Viral Tropism, Viral Entry, Microbiome, Gastroenteritis, microfluidics

## Abstract

Noroviruses are the leading cause of acute gastroenteritis around the world. An individual living in the United States is estimated to develop norovirus infection five times in his or her lifetime. Despite this, there is currently no antiviral or vaccine to combat the infection, in large part because of the historical lack of cell culture and small animal models. However, the last few years of norovirus research were marked by a number of ground-breaking advances that have overcome technical barriers and uncovered novel aspects of norovirus biology. Foremost among them was the development of two different *in vitro* culture systems for human noroviruses. Underappreciated was the notion that noroviruses infect cells of the immune system as well as epithelial cells within the gastrointestinal tract and that human norovirus infection of enterocytes requires or is promoted by the presence of bile acids. Furthermore, two proteinaceous receptors are now recognized for murine norovirus, marking the first discovery of a functional receptor for any norovirus. Recent work further points to a role for certain bacteria, including those found in the gut microbiome, as potential modulators of norovirus infection in the host, emphasizing the importance of interactions with organisms from other kingdoms of life for viral pathogenesis. Lastly, we will highlight the adaptation of drop-based microfluidics to norovirus research, as this technology has the potential to reveal novel insights into virus evolution. This review aims to summarize these new findings while also including possible future directions.

## Introduction

Noroviruses form a genus within the *Caliciviridae* family that is subdivided into seven genogroups (G) and more than 30 genotypes ^1^. These non-enveloped viruses have a positive-sense, single-stranded RNA genome ^~^7.7 kb in size that is typically organized into three open reading frames (ORFs) ^2^. ORF1 encodes the non-structural proteins; ORF2 encodes VP1, the major capsid protein; and ORF3 encodes VP2, the minor capsid protein ^3^. Murine noroviruses also have a fourth ORF, which encodes an antagonist of the innate immune response called virulence factor 1 (VF1) ^4^. The major capsid protein VP1 forms virions and is divided in two domains: the shell (S) domain, which encases the viral RNA, and the protruding (P) domain ^5^. The P domain interacts with the carbohydrate attachment receptors, contains neutralizing epitopes, and evolves under immune selection pressure ^6– 8^.

Human noroviruses are the leading etiologic agent of viral gastroenteritis globally in people of all ages ^9, 10^, costing ^~^4 billion US dollars in direct healthcare costs and ^~^60 billion US dollars in societal costs (e.g. lost productivity) worldwide ^11^. These infections cause an estimated 200,000 deaths annually in children under 5 years of age in developing countries ^12^. In the US alone, human noroviruses are estimated to annually cause 19–21 million infections and cost ^~^2 billion US dollars/year ^13, 14^. The majority of these infections are caused by GII, genotype 4 viruses (GII.4) ^15^. However, no directed disease prevention and treatment modalities are available. This is in part because of historical limitations in working with these viruses in the laboratory. However, recent breakthroughs are overcoming previous challenges: for example, the development of two *in vitro* culture systems ^16, 17^, a mouse model ^18^ and the identification of functional receptors for murine norovirus ^19, 20^. This review aims to summarize current knowledge about norovirus tropism, cellular entry, interactions with commensal bacteria, and the application of drop-based microfluidics to the analysis of viral evolution ^21– 24^. Owing to length restrictions, we are unable to cover all of the exciting advances in the norovirus field. However, the reader is referred to other excellent recent reviews for examples of norovirus replication ^25^, norovirus antiviral and vaccine development ^26– 28^, and norovirus epidemiology and disease burden ^29^.

## Norovirus cell tropism

Norovirus cell tropism has long been debated in the field ^30^. The first culture system for a norovirus was described for murine norovirus in murine macrophages and dendritic cells following the observation that these cell types were infected in STAT1-deficient mice *in vivo* ^31^. Subsequent work demonstrated an additional tropism of murine norovirus for murine B cells ^16^, the extent of which is determined by residues in the VP1 P domain ^32^. An immune cell tropism for human norovirus was also observed in some animal models ^18, 33^. While attempts at culturing human noroviruses in blood-derived macrophages and dendritic cells were unsuccessful ^34^, some human B cell lines (BJAB, Raji, Namalwa) support infection with one strain (GII.4 Sydney) of human norovirus ^16, 35^. Additional studies are underway to test the susceptibility of these cell lines to additional human norovirus strains and genotypes. Nevertheless, subsequent work demonstrated the applicability of this first *in vitro* culture system for efficacy studies of human norovirus antivirals ^36^, highlighting the power of this system for anti-norovirus drug development. In addition to the observed human norovirus infection of immune cells, an intestinal epithelial cell tropism of human norovirus has long been predicted, given the striking gastrointestinal symptoms and described perturbations of epithelial cells ^37– 40^. However, previous attempts to cultivate human norovirus in intestinal epithelial cells *in vitro* were unsuccessful ^41– 44^. In contrast, a recent breakthrough describes the use of stem-cell-derived intestinal enteroids as another *in vitro* culture system for human noroviruses ^17^. Enteroid cultures derived from stem cells from the duodenum, jejunum, or ileum are susceptible to human norovirus infection when the cells are matured into a monolayer. Unlike BJAB B cells ^16^, human norovirus-infected intestinal epithelial cells exhibit cytopathic effects ^17^. VP1 capsid protein staining revealed that specifically enterocytes, but not goblet cells or enteroendocrine cells, are infected. Interestingly, treatment of monolayers with bile acids was required for infection by some strains (e.g. GII.3), while it was not required but did improve infection by other strains (e.g. GII.4). Great variability was observed in overall viral titers between different strains and isolates ranging from 10–1000-fold. Viral titers typically increase ^~^10–300-fold in either *in vitro* culture system—thus, a direct comparison of the same virus stock in both systems is needed to determine whether one system supports greater replication levels than the other. The strength of the BJAB system lies in its simplicity and ease of use once established. However, further efforts are needed to overcome the variabilities seen between laboratories ^35^. While the enteroid system is known to support the replication of multiple human norovirus strains and isolates, establishment and use of the system is costly and time intensive. Nevertheless, the exciting discovery of two human norovirus *in vitro* culture systems provides a technological advance for much-needed basic and translational studies in the future.

The dual tropism of human norovirus for B cells and enterocytes *in vitro* raises questions about the nature of the human norovirus-infected cell types *in vivo*. Intriguing new data obtained from immunostained small intestinal sections of human norovirus-infected patients ^45^ are consistent with a dual tropism for immune cells and intestinal epithelial cells. Specifically, these data demonstrate the expression of structural and/or nonstructural proteins in cells positive for CD68 or DC-SIGN (i.e. macrophages and dendritic cells), CD3 (i.e. intraepithelial lymphocytes and T cells), and villin (i.e. enterocytes). This tropism for multiple cell types is also reminiscent of bovine norovirus, where capsid antigen was detected in villus enterocytes and in lamina propria immune cells ^46^. The temporal expression of bovine norovirus antigen, first in epithelial cells and subsequently in immune cells ^46^, as well as detection of the epithelial marker cytokeratin 8 in human norovirus-infected macrophages ^45^ were interpreted as evidence for phagocytosis of infected epithelial cells by antigen-presenting cells. However, given the normal phagocytic function of these cells, direct viral infection of antigen-presenting cells cannot be ruled out. Unfortunately, the question of whether human B cells are infected *in vivo* could not be addressed since CD20+ B cells were not detectable in the histologic sections of human norovirus-infected immunodeficient patients ^45^. However, the finding that human SCID patients who lack B cells are able to be infected with human norovirus is consistent with a tropism for multiple cell types, including B cells ^47^. The viral titer of these patients was approximately one log lower compared to patients with B cells, suggesting that B cells contribute to the overall viral loads. Taken together, B cells, in addition to enterocytes, are an important player in human norovirus pathogenesis, although their precise contribution and viral factors in that process remain to be elucidated. For murine norovirus, the interaction with B cells is multi-faceted. Early in infection, the murine norovirus minor capsid protein VP2 antagonizes B cell antigen presentation to CD8 cytotoxic T cells in a strain-specific manner ^32^. Later in infection, antibody is critical for the clearance of murine norovirus infection ^48^. Further studies are clearly required to elucidate the role of each target cell type and the temporal pattern of infection during norovirus pathogenesis, as well as the contribution of viral factors to any phenotype. Furthermore, the susceptibility of enterocytes to murine norovirus should be revisited.

## Norovirus binding and entry

In general, viral infection of susceptible target cells is a multistep process that begins with the binding of viral particles to attachment receptors, which helps to concentrate the particles on the cell surface ^49^. Subsequent interaction with additional receptor molecules (e.g. functional or entry receptors) actively promotes virus uptake: for example, by initiating conformational changes in the virus capsid, activating cellular signaling, promoting endocytosis, or directly driving penetration ^49, 50^. Although little is known about norovirus entry, we hypothesize that the entry process is likely characterized by similar features.

The attachment receptors for most human noroviruses are histo-blood group antigens (HBGAs), terminal complex carbohydrates of lipid- or protein-linked glycan chains ^51– 53^. HBGAs are expressed on multiple cells types, including intestinal epithelial cells, and secreted into body fluids, such as saliva ^51, 54^. Two to four binding sites within the human norovirus P dimer bind to α1,2-linked fucose on HBGAs, a residue attached to the carbohydrate core by the α(1,2) fucosyltransferase (FUT2) enzyme ^55– 59^. Individuals with a functional FUT2 enzyme are termed secretors, while those without are known as non-secretors. Secretors are more susceptible to many human norovirus strains (e.g. GII.4) and other diarrheal diseases ^60– 63^. In culture, GII.4 strains of human norovirus infect enteroids derived from secretor but not non-secretor individuals ^17^, suggesting that susceptibility to norovirus infection is determined at the level of the cell. One potential mechanism of promoting infection may be by enhancing cell attachment, since synthetic HBGAs or enteric bacteria expressing HBGA-like structures increase viral attachment to B cells ^16^. However, the precise role(s) that specific HBGAs play in different locations in the body, or when attached to different cores from host or enteric bacteria, remains to be addressed in future studies. Studies to date have investigated HBGA-linked lipids. Glycosphingolipids (GSLs), including those containing HBGA, are a main constituent of the plasma membrane of many cells. The ceramide base anchors GSLs to the membrane, while the glycan group extends into the extracellular space ^64^. GSLs serve as attachment receptors for multiple non-enveloped viruses (e.g. rotavirus and polyomavirus) ^65^. Depending on the strain, human norovirus virus-like particles (VLPs) can bind to the HBGA groups of GSL ^66^ but also to the galactose-containing GSL galactosylceramide ^67^ or gangliosides ^68^, which are sialic-acid-containing GSLs. Similarly, murine norovirus binds to gangliosides on murine macrophages, specifically the terminal sialic acid of GD1a, in the case of the MNV-1 strain ^69^. The interaction between human norovirus particles and the cellular membrane GSLs occurs only above a certain threshold concentration of HBGA-GSL and in a virus strain-specific manner ^70^. This interaction then clusters the GSLs to form a lipid microdomain and causes invagination of that membrane region ^71^. The downstream events of human norovirus cellular entry are still unknown and await further study in the recently developed cell culture systems ^16, 17^. Endocytosis of murine norovirus particles is dependent on dynamin II, ceramide, and cholesterol but is independent of clathrin, caveolin, and pH ^72– 74^. Ceramide is the backbone of GSL structures ^75^, while cholesterol stabilizes lipid microdomains ^76^. Therefore, it is possible that, like murine norovirus, human norovirus entry will also depend on these two factors. Interestingly, human norovirus infection is dependent on, or enhanced by, bile acids in enteroid cultures ^17^. Bile acids can directly induce ceramide production by activating the farnesoid X receptor (FXR) ^77, 78^, a nuclear receptor expressed in many tissues, including in the intestine and immune cells ^79, 80^. FXR induction, in turn, increases the expression of enzymes involved in the production of ceramide, including Smdp3 (neutral sphingomyelinase II) and Smpd4 (neutral sphingomyelinase III) ^78^, which form ceramide after hydrolyzing sphingomyelin ^81^. Ceramide then transactivates glucosylceramide synthase expression, which generates glucosylceramide, the starting point for GSL biosynthesis ^82, 83^. A larger quantity of ceramide in a cell may thus facilitate the production of GSL attachment receptors for noroviruses, providing a potential explanation for the dependence of these viruses on bile acids or ceramide.

Other recent studies have described the discovery of multiple proteins involved in murine norovirus binding and entry. CD36, CD98, transferrin receptor, and CD44 are cell-type-specific modulators of murine norovirus infection in murine macrophages and murine dendritic cells, though the mechanism by which these proteins aid in infection awaits further study ^84^. An exciting discovery was the identification of CD300lf and CD300ld as the murine norovirus entry receptors ^19, 20^, the first and, to date, only functional receptors known in the norovirus genus. CD300lf was identified as a critical protein during murine norovirus infection in CRISPR-Cas9 screens in BV2 ^20^ and RAW264.7 cells ^19^. The murine norovirus binding site on CD300lf was mapped to amino acids 39 to 45 at the protein’s N-terminus. This is a region completely conserved in the related family member CD300ld, providing an explanation for the ability of murine norovirus to bind to both molecules ^19^. Transfection of murine *CD300lf* or *CD300ld* into non-susceptible but permissive HeLa and HEK293T human cell lines rendered these cells susceptible to murine norovirus infection ^19, 20^. However, transfection of human *CD300lf* into murine cells did not mediate murine norovirus susceptibility ^20^, suggesting that CD300lf is a receptor determining both cell tropism and species specificity. Whether human CD300lf or CD300ld serve as a receptor for human norovirus remains to be determined. CD300 proteins, like the only other known functional receptor in the *Caliciviridae* family, JAM-A, are part of the Ig superfamily of proteins ^85, 86^. Clustering in lipid raft domains is essential for CD300 protein function ^87^, providing a potential interaction platform between norovirus attachment and functional receptors at the cellular level. *In vivo, Cd300lf* deletion renders mice resistant to shedding following oral murine norovirus infection ^20^. However, the precise role of CD300lf during pathogenesis remains to be investigated. Expression of CD300lf can be induced by conditions known to induce microfold (M) cell differentiation ^88^. Since these cells are important for murine norovirus transcytosis across intestinal epithelial layers *in vitro* and *in vivo* ^89– 91^, this suggests CD300lf may function in a cell-type-specific manner. CD300ld regulates the expression of other CD300 molecules ^85^ and stimulates macrophages to secrete pro-inflammatory cytokines and chemokines ^92^. In macrophages, antibody cross-linking of CD300ld increases the secretion of TNF-α and IL-6 ^93^, two pro-inflammatory cytokines also upregulated during human norovirus infection *in vivo* ^94, 95^. Future investigations are needed to investigate the roles of CD300lf and/or CD300ld in species specificity of norovirus infection, norovirus cellular and tissue tropism, and murine norovirus transcytosis through M cells and pathogenesis.

Taken together, one possible model of norovirus entry might be as follows. Norovirus virions bind to one or several GSLs on the host cell surface, allowing for viral adhesion to and movement along the fluid surface of the plasma membrane. The multivalent particles then bind more GSLs, which are present in already formed lipid domains, such as lipid rafts, or newly form a cluster of lipids at the interaction site between the virus particle and the cell membrane. Transmembrane proteins such as CD300 proteins ^87^ may already be resident in lipid rafts or may be recruited to the microdomain induced by norovirus particle binding. This could create a stable, cholesterol-dependent platform for noroviruses to promote engagement with signaling molecules and the functional receptor. The multivalency of the norovirus capsid in concert with GSLs may cause invagination of the plasma membrane ^71^. Norovirus-containing vesicles may next be released from the plasma membrane by dynamin II-mediated scission of the invagination, followed in quick succession by viral genome release into the cytosol ^73^. Although the past 10 years have shed light on some aspects of norovirus entry, much still remains to be addressed, such as the interplay between different norovirus receptors, potential conformational changes in the virus capsid, potential activation of cellular signaling, and the mechanism of membrane penetration.

## Norovirus and the intestinal microbiome

Recent studies have led to an appreciation that infection by enteric viruses, including noroviruses, is influenced by the commensal microbiota ^16, 96, 97^. While a mechanistic understanding of these transkingdom encounters is still lacking, the proviral or antiviral functions of the microbiota are both direct and indirect ^98^. In the case of noroviruses, one example for a direct effect is the enhancement of GII.4 human norovirus infection of B cells by the commensal bacterium SENG-6, an *Enterobacter cloacae* strain ^16^. This effect is mediated, at least in part, by increasing cell attachment via bacterially expressed HBGA-like molecules. Other bacterial species similarly express HBGA-like structures ^99^ and thus might also be able to stimulate infection *in vitro*, although this still needs to be tested experimentally in both culture systems. The opposite was observed *in vivo* during infection of gnotobiotic pigs, where *E. cloacae* inhibited human norovirus infection, compared to uncolonized pigs ^100^. However, the study lacked an important control (namely, a bacterial strain unable to bind human norovirus) and thus the effect of *E. cloacae* on norovirus infection *in vivo* remains unclear. Another proviral direct effect of bacteria during norovirus infection is via increasing particle stability and protecting virions from heat stress ^99^. This may promote viral transmission and environmental fitness similar to poliovirus ^101^, but future studies are required to directly test this.

Indirect mechanisms are thought to occur via modulation of the antiviral immune response by the microbiota. This is consistent with the observed increase in murine norovirus loads in the ileum of conventional mice compared to antibiotic-treated or germ-free mice ^16, 102^ and the type III interferon-mediated changes in murine norovirus persistence ^103^. In contrast to the proviral effects of the microbiota, at least one bacterial genus, *Lactobacillus,* can also play a protective role against norovirus infections. A higher abundance of *Lactobacillus* due to probiotic-fermented milk ingestion correlates with a quicker recovery from human norovirus-induced fever ^104^. Similarly, a higher abundance of *Lactobacillus* following experimental vitamin A treatment correlates with inhibition of murine norovirus infection ^105^. These initial observations suggest that the microbiota and its members can be either protective or stimulatory for norovirus infections. Another indirect effect may also occur through the modulation of glycan molecules that mediate viral attachment. Specifically, intestinal colonization with the microbiota upregulates *fut2* expression via ERK and JNK signaling cascades ^106^, while colonization with CagA-positive *Helicobacter pylori* correlates with α(1,2) fucosylated epitopes on intestinal surfaces ^107^. Since an individual’s secretor status influences their microbiota composition ^108, 109^, there appears to be a reciprocal relationship between the host and bacteria that determine the overall level of fucosylation in the intestine ^110^. The modulation of receptor binding sites may also extend to proteinaceous receptors. The murine norovirus receptor CD300lf is upregulated by lipopolysaccharide (LPS) stimulation ^111, 112^. Thus, Gram-negative bacteria might upregulate this viral receptor. Engagement of CD300lf can also upregulate TNF-α expression ^113^, which may explain the observed TNF-α increase during human and murine norovirus infection ^94, 114, 115^. Interestingly, TNF-α induces sialyltransferase activity ^116, 117^ as well as lactosylceramide synthase activity, an enzyme that produces lactosylceramide from glucosylceramide, a precursor for most glycosphingolipids ^118^. Therefore, engagement of inflammatory pathways may upregulate gangliosides and sialic acids on the cell surface, known murine norovirus attachment factors ^69, 119^. This might provide an explanation for why murine norovirus does not share the same level of dependence on the presence of bile acids as do human noroviruses ^17, 74^. Yet both viruses may have evolved ways to promote upregulation of host receptor molecules to facilitate infection. Clearly, more studies are needed to investigate these potential links and gain a mechanistic understanding of the multifaceted role of the microbiota on norovirus infections.

## Microfluidics in norovirus research

An exciting technical development in the field is the application of drop-based microfluidics to norovirus research. This ultrahigh-throughput platform uses aqueous drops dispersed in oil as picoliter-volume reaction vessels to screen and analyze single cells or individual virus particles at a rate and level of sensitivity that are far superior to the traditional cell-culture-based techniques used currently, such as plaque assay, and other biochemical-, genetic-, and molecular-based techniques ^21, 22, 120– 124^. The production of millions of drops that are amenable to high-speed measurements in parallel reduces cost and screening time ^125, 126^. Furthermore, microfluidics overcome the loss of error due to the presence of minor alleles associated with bulk viral culture, since each encapsulated virus particle constitutes an independent experiment, facilitating viral evolution studies. Over the past five years, microfluidics has been successfully used to i) grow murine norovirus to identify mutations that support escape from neutralizing monoclonal antibody A6.2 ^22^, ii) develop a rapid, targeted, and culture-free infectivity assay to determine the efficacy of a neutralizing antibody for murine norovirus ^21^ with comparable results to plaque-based neutralization assays, iii) detect, quantify, and sequence artifact-free rare recombinant noroviruses *in vitro* ^24^ and *in vivo* ^23^, providing critical information that cannot be obtained using traditional methods such as phylogenetic studies ^127, 128^ and full genome sequencing ^128^, and, lastly, iv) simultaneously screen for multiple viruses, including noroviruses, in environmental water samples ^129, 130^. The microfluidics technology is continually being improved and adapted to a wider array of scientific inquiries ^121^ and has the potential to become an integral platform for future norovirus research, specifically aiding in studies of norovirus evolution and population dynamics, drug screening, and environmental testing.

## Conclusion

Noroviruses have proven difficult to study in the past, but progress in overcoming technical barriers has opened doors to much-needed basic and translational studies. The recent development of two new culture systems has already yielded new biological findings about the role of bile acids and HBGAs in norovirus infection ^16, 17^. Further refinement of each system and improvements in ease of use and reproducibility will increase their utility to the scientific community and facilitate new discoveries. In addition, the identification of multiple norovirus-infected cell types of both epithelial (i.e. enterocytes) and immunological (e.g. B cells, macrophages, dendritic cells, and T cells) origin represents a shift in the paradigm from the assumed enterocyte-tropic nature of noroviruses to a more inclusive definition of an enterotropic virus: one that encompasses infection of multiple cell types residing in the intestine. Therefore, culture systems that recapitulate infection of each cell type will advance a greater understanding of the cell-type-specific vs. general mechanisms that drive norovirus infection. At the same time, the appreciation of this broader tropism also suggests that more advanced co-culture systems encompassing multiple cell types will be needed to dissect the interactions between norovirus and the various cell types and their specific roles in pathogenesis. In addition, early findings regarding the importance of intestinal microbiota and norovirus encounters on the outcome of infection provide an exciting new direction in the norovirus field, revealing interesting details about the complex interplay between highly different organisms. Combining the expertise of scientists within and outside the norovirus field to take advantage of new methodologies such as microfluidics will further increase our understanding of norovirus biology and our ability to develop effective solutions for infection prevention, treatment, and control.
